# Epidemiological Dynamics of Extended-Spectrum *β*-Lactamase- or AmpC *β*-Lactamase-Producing *Escherichia coli* Screened in Apparently Healthy Chickens in Uganda

**DOI:** 10.1155/2021/3258059

**Published:** 2021-09-20

**Authors:** Steven Kakooza, Damien Munyiirwa, Paul Ssajjakambwe, Edrine Kayaga, Dickson Stuart Tayebwa, Dickson Ndoboli, Loreen Basemera, Esther Nabatta, Maria Agnes Tumwebaze, John Baligwamunsi Kaneene

**Affiliations:** ^1^Central Diagnostic Laboratory, College of Veterinary Medicine, Animal Resources and Bio-Security Makerere University, Kampala, Uganda; ^2^Vétérinaires Sans Frontières Germany, Kampala, Uganda; ^3^Novel Minds Science Plexus, Kampala, Uganda; ^4^Department of Veterinary Pharmacy, Clinics and Comparative Medicine, College of Veterinary Medicine, Animal Resources and Bio-Security Makerere University, Kampala, Uganda; ^5^Infectious Diseases Institute, College of Health Sciences Makerere University, Kampala, Uganda; ^6^Michigan State University, Center for Comparative Epidemiology, 736 Wilson Road, Room A-109, East Lansing, MI 48824, USA

## Abstract

The dynamics of extended-spectrum *β*-lactamase- (ESBL-) and AmpC *β*-lactamase-producing bacteria (which are deadly groups of antimicrobial-resistant bacteria) have not been well understood in developing countries. This raises major concerns to antimicrobial resistance (AMR) control. We investigated the prevalence and factors linked to the fecal carriage of ESBL- or AmpC-producing *Escherichia coli* (ESBL-/AmpC-EC) in commercial chickens. Cloacal swabs from 400 birds were sampled and submitted to the Central Diagnostic Laboratory for ESBL-/AmpC-EC screening by culture methods using MacConkey agar supplemented with cefotaxime. Epidemiological data were collected using a structured questionnaire and plausible risk factor analyses prepared by R software using *X*^2^ test and logistic regression modeling. Results showed that the prevalence of ESBL-/AmpC-EC was 17.5%. Univariable screening hypothesized that carriage was probably influenced by a type of commercial chicken, geographical location, age group, flock size, and housing system (*p* < 0.05). Modeling exposed that broiler birds were at a higher risk of being ESBL-/AmpC-EC carriers (COR = 9.82, CI = 3.85–25.07). Birds from Wakiso Town Council (COR = 4.89, CI = 2.04–11.72) and flocks of 700–1200 birds were also at a higher risk of harboring ESBL-/AmpC-EC (COR = 2.41, CI = 1.11–5.23). Birds aged 4 months and below were more susceptible to ESBL-/AmpC-EC carriage compared with those aged 1 month and below being 6.33 times (CI = 1.65–24.35) likely to be carriers. The occurrence of ESBL-/AmpC-EC in flocks suggests possible treatment failures while managing colibacillosis. Consequently, injudicious antimicrobial use should be replaced with an accurate diagnosis by bacterial culture and sensitivity testing so as to circumvent AMR emergence, spread, and associated losses.

## 1. Introduction

Although antimicrobial resistance (AMR) is an ancient phenomenon, the burden of extended-spectrum *β*-lactamase- (ESBL-) and AmpC-producing bacteria in animals (particularly poultry) became significantly higher after the usage of *β*-lactam antibiotics [1]. The high bacterial exposure to *β*-lactam antibiotics in poultry farming, usually through feed additives and clinical or prophylactic treatments, leads to the exclusion of sensitive strains but spares the resistant mutant ones. ESBL and AmpC enzymes have the ability to hydrolyze a number of *β*-lactam antimicrobials such as cephalosporins and monobactams [2]. In the long run, this increases the difficulty of treating infections, especially in the human sector where *β*-lactams are more used. In Uganda, reports which expose antimicrobial resistance effects on production and farmers' livelihoods exist, particularly the increasing prevalence of treatment failures and deaths (mediated by resistant strains). This problem could be attributed to the insensible use of antibiotics by Ugandan farmers. Currently, antimicrobials are also readily acquired by many over the counter because regulations to limit access to veterinary drugs are liberal, in that way sponsoring the use of these drugs by poultry farmers indiscriminately in production. In addition to some of the popular drug classes such as tetracyclines (oxytetracyclines), quinolones (enrofloxacin), potentiated sulphonamides (trimethoprim/sulfamethoxazole), aminoglycosides (neomycin and gentamicin), and macrolides (tylosin), worrying reports also show the use of highly critical human drugs such as antiretrovirals in poultry production [3]. There is no clear evidence on the use of certain *β*-lactam classes such as cephalosporins, carbapenems, carbacephems, and monobactams in poultry. The most commonly used *β*-lactam class on the market is the penicillins (such as ampicillin and amoxicillin). The majority of the ESBL- and AmpC-producing bacteria come from members in the Enterobacteriaceae family [4], such as *Escherichia coli* (*E. coli*) and *Klebsiella pneumoniae*. The latter bugs are currently considered as emerging zoonotic and multidrug-resistant bacteria, criticized to pose a major challenge in antimicrobial treatments [5, 6]. Human exposure to such lethal bacteria can be through consumption of contaminated poultry products such as meat and eggs [7] with most of these fowl-hosted strains ably causing disease in humans [8]. This renders AMR in food animals a public health threat requiring joint coordinated efforts [9].

Reservoirs and transmission routes of ESBL and AmpC bacteria from animals to humans in the community remain not fully explored [10]. Though poultry is taken as one of the major reservoirs of antimicrobial-resistant bacteria [5], limited findings on some categories of antimicrobial-resistant bacteria (ARB) such as extended-spectrum *β*-lactamase- and AmpC-producing *E. coli* still exist. The geographical distribution of ESBL and AmpC *E. coli* in poultry differs with some studies portraying worrying prevalence rates of over 20% in chickens [2, 11–14]. Their information is not clear on the interplay between the microbes, their earmarked hosts, and the aspects stimulating carriage and dissemination. This glaring gap also exists in the poultry industry (especially chicken in Uganda), a challenge to AMR control measures. Therefore, this study sought to better understand the dynamics of ESBL- or AmpC *β*-lactamase- (ESBL-/AmpC-) producing bacteria in selected chicken farms focusing on prevalence and drivers for ESBL/AmpC *E. coli* (ESBL-/AmpC-EC) carriage. Emphasis was on commercial intensive systems in Wakiso district, Uganda.

## 2. Materials and Methods

### 2.1. Study Design and Area

A cross-sectional study was carried out between November 2019 and April 2020 in the Wakiso district (00°24′N 32°29′E) of Central Uganda. We purposely selected the district since it is one of the key local hubs with a high number of commercial poultry farms [15].

### 2.2. Sampling and Data Collection

Epidemiological sample size calculations took into account recommendations by the documentation of Wang and Cheng [16]. An estimated prevalence of 50% carriage of ESBL-/AmpC-EC was used at a confidence level of 95% and precision of 0.05. Having calculated a total of 384 samples, 400 cloacal swabs were collected from 20 poultry farms (20 samples each). Poultry management systems in Uganda were categorized into intensive, semi-intensive, and extensive. In this study, we concentrated on intensive farms since they are more popular [17] and a high consumer of antibiotics which increases the risk of birds carrying resistant bacteria [12]. The inclusion criteria excluded farms with less than 100 birds. According to the District Veterinary Officer (DVO), the district has more than 50 intensive poultry farms, but the list was influenced by farmers rearing chicken at that time, consent to participate in the surveillance, and the ease of access to the location. Data collected included type of commercial poultry (broilers or layers or dual purpose), age of the birds, current health status of the birds, use of antimicrobials (particularly, *β*-lactam antibiotic derivatives), flock sizes, geographical location, and the housing system.

### 2.3. Screening of ESBL-/AmpC-EC

Among the Enterobacteriaceae family, we focused on *E. coli*. We screened for ESBL-/AmpC-EC according to the methods by [18–20]. Cloacal swabs were pre-enriched in MacConkey broth (Conda, Spain) supplemented with 1 mg/L of cefotaxime [21] and incubated at 37°C for 24 hours. The overnight cultures were then streaked on MacConkey agar (Conda, Spain) supplemented with 1 mg/L of cefotaxime prior to incubation at 37°C for 24 hours. Pink colonies surrounded by a zone of precipitation of bile salts were again subcultured on the same first isolation agar. The colonies were biochemically identified as *E. coli* using indole, methyl red, urease, Voges–Proskauer, citrate, and lactose utilization tests [8, 22].

### 2.4. Data Analysis

Data were entered and cleaned in Microsoft Excel (version 2019) prior to importation into Statistical Package for Social Sciences (SPSS) (version 25.0) and R statistical software for analysis. Descriptive statistics were used to summarize the data, and statistics were presented as frequencies and percentages. The prevalence of ESBL-/AmpC-EC was calculated as the proportion of the positive cases to the number of birds tested in the study. A positive ESBL-/AmpC-EC status was defined as the detection of at least one ESBL-/AmpC-EC isolate in the sample cultured. The corresponding confidence intervals of prevalence were computed as exact binomial 95% confidence intervals using a calculator from https://sample-size.net/confidence-interval-proportion/. The chi-square (*X*^2^) test was also used to evaluate significant differences (*p* < 0.05) between the prevalence of ESBL/AmpC-EC and the variables (type of commercial poultry, subcounty, housing system, flock size, and age group). For the probable risk factor analyses, the outcome investigated was ESBL-/AmpC-EC status for each bird. Potential risk factors (*p* < 0.05) from the *X*^2^ test were sorted for inclusion in a bivariate regression model. The value of the *X*^2^ test was also used to ascertain for associations between pairs of variables (housing system, deep litter, or battery cage). Variables with *p* < 0.05 in the model were considered factors for ESBL-/AmpC-EC carriage. Model goodness-of-fit tests were also performed.

### 2.5. Ethical Considerations

Farm owners or managers were given consent forms prior to inclusion in the study. The forms explained the study and stipulated the roles of contributing farms and benefits from participation in the research.

## 3. Results

### 3.1. Poultry Sample Demographics

Cloacal swabs were sampled from 400 chickens reared on 20 farms located in different sub-counties (Kakiri-100; Kasangati Town Council-100; Kyengera Town Council-100; Mende-40; Wakiso-20; and Wakiso Town Council-40) of the Wakiso district. A proportion of 60% (240/400) were reared for meat (broilers), 35% (140/400) for eggs (layers), and 5% (20/400) for both meat and eggs (dual purpose). The mean, range, and median of the chicken flock sizes on the visited farms were 2,201.0 (*n* = 20), 170–13,000, and 1,250.0, respectively. The samples were equally distributed between farms having over 1,200 (200/400) and those below 1,200 (200/400) birds. The mean, range, and median of the birds' ages were 3.8 (*n* = 400), 0.5–14, and 1.5 months, respectively. 60% (240/400) were below 5 months old, and 40% (160/400) were 5 months old and above. The majority of the samples were from the deep litter housing system birds (95%, 380/400) and 5% (20/400) from the battery cage birds (Table 1). At the time of visit, none of the flocks had clinical signs of the disease and were being given antibiotics, particularly *β*-lactams. However, three farms confirmed using antibiotics and growth promoters in the past 3 months; one was a broiler farm which had used enrofloxacin (quinolone), Vetgrow (growth promoter), and oxytetracycline (tetracycline), a layer farm that had used tylosin (macrolide) and oxytetracycline (tetracycline), and another broiler farm that had used vitamins.

### 3.2. Prevalence of ESBL-/AmpC-EC

A total of 70 out of 400 samples (17.5%; CI = 13.9–21.6) tested positive for ESBL-/AmpC-EC. The prevalence by the type of commercial poultry was 26.7% (64/240; CI = 21.2–32.7) in broilers, 3.6% (5/140; CI = 1.2–8.1) in layers, and 5.0% (1/20; CI = 0.1–24.9) in dual purpose, respectively. There was a significant difference in the prevalence of ESBL-/AmpC-EC amongst various types of commercial chicken (*X*^2^ = 34.95, df = 2, and *p* < 0.001). The prevalence in different subcounties was 12.0% (12/100; CI = 6.4–20.0), 18.0% (18/100; CI = 11.0–27.0), 14.0% (14/100; CI = 7.9–22.4), 22.5% (9/40; CI = 10.8–38.5), 5.0% (1/20; CI = 0.1–24.9), and 40.0% (16/40; CI = 24.9–56.7) in Kakiri, Kasangati Town Council, Kyengera Town Council, Mende, Wakiso, and Wakiso Town Council, respectively. There was a significant difference in the prevalence of ESBL-/AmpC-EC amongst various geographical farm locations (*X*^2^ = 19.84, df = 5, and *p*=0.001). The prevalence by the type of poultry housing system was 18.4% (70/380; CI = 14.7–22.7) in deep litter birds and 0% (0/20; CI = 0.0–16.8) in battery cage birds. There was a significant association between the prevalence of ESBL-/AmpC-EC and the housing system (*X*^2^ = 4.47, df = 1, and *p*=0.035). The prevalence by age group was 25% (11/44; CI = 13.2–40.3), 21.9% (43/196; CI = 16.4–28.4), 13.0% (13/100; CI = 7.1–21.2), and 5% (3/60; CI = 1.0–13.9) in birds <1 month, 1–4 months, 5–8 months, and >8 months old. There was a significant difference in the prevalence of ESBL-/AmpC-EC amongst various age groups (*X*^2^ = 12.29, df = 3, and *p*=0.006). The prevalence was 24.3% (34/140; CI = 17.4–32.3), 25.0% (15/60; CI = 14.7–37.9), 6.7% (4/60; CI = 1.9–16.2), and 12.1% (17/140; CI = 7.2–18.7) in birds sampled from flocks of <700, 700–1,200, 1,201–1,701, and >1,701 birds, respectively. There was a significant association between the prevalence of ESBL-/AmpC-EC and flock sizes where the tested samples came from (*X*^*2*^ = 14.46, df = 3, and *p*=0.002).

### 3.3. Predictors for ESBL-/AmpC-EC Carriage in Commercial Chickens

#### 3.3.1. Bivariate Logistic Regression

The risk of ESBL-/AmpC-EC carriage was increased in broilers (COR = 9.82, CI = 3.85–25.07). Samples from Wakiso Town Council were 4.89 times likely to test positive for ESBL-/AmpC-EC. The risk of having ESBL-/AmpC-EC was more pronounced amongst birds less than 1 month old (COR = 6.33, CI = 1.65–24.35). Birds from flock sizes of 700 to 1,200 were at a higher risk of harboring ESBL-EC (COR = 2.41, CI = 1.11–5.23).

## 4. Discussion

Although ESBL-/AmpC *β*-lactamase-producing bacteria have been implicated as a threat in the society, their addition in National Antimicrobial Resistance Monitoring Systems is still lacking in developing countries, Uganda inclusive. Deficiencies in diagnostic testing contribute to a paucity of the AMR data essential in policy formulation and implementation. There is a lack of a standard method for the detection of ESBL/AmpC *β*-lactamases [19]; however, several methods are in use for screening. Using a conventional screening approach, we give a baseline picture of ESBL-/AmpC-producing bacteria in chicken flocks to motivate monitoring efforts in Uganda's food-producing animals.

The study reports an overall prevalence of ESBL-/AmpC-EC of 17.5%, which was lower when compared to a range of 20% to 87% compiled from other studies [11–13, 23]. The variations in prevalence rates could be the difference in ESBL/AmpC diagnostic methods used. In addition, marginal use of *β*-lactam antibiotics was noted on the farms. The occurrence of noxious groups of ARB in flocks may question the safety of poultry products, especially in countries such as Uganda with nonstringent food safety systems [8]. From the farm, a critical meat contamination node could be the slaughter house [19] thus standardizing chicken meat handling processes and supporting their operationalization which must be prioritized to control the contamination of consumer products.

The distribution of ESBL/AmpC bacteria in different geographical locations has been known to vary [5]. This was confirmed in this study as farm samples from Wakiso Town Council were more likely to test positive for ESBL-/AmpC-EC. This could be attributed towards the diversity in management practices done in various areas. Also, the role of human population density (driven by majorly urbanization) could be an underlying driver as humans are known reservoirs of ARB [24]. Wakiso Town Council had more human settlements compared to the other subcounties (Kakooza, personal observation).

Poultry in Uganda are reared in different production arrangements: extensive (free range), intensive, and semi-intensive systems [17]. Our study focused on intensive under 2 housing systems, that is, deep litter and battery cage management. Samples from the deep litter system had a higher ESBL-/AmpC-EC prevalence compared to those from the battery cage system. This inference could be a systemic bias as broiler birds (which had higher ESBL-/AmpC-EC prevalence rate) were mainly reared in deep litter systems.

The probable risk of occurrence of ESBL-/AmpC-EC was increased with broiler birds more than in layer and dual-purpose birds. This finding was in agreement with results from a study conducted by Brower et al. [11]. Broilers are known for their high feed intake, and this behaviour is a stimulus for continuous shedding of feces, thus increasing the risk of their environmental contamination with different bacterial strains [8]. Additionally, many broiler farms had high stock densities which could make environmental management aimed at lowering bacteria in the houses more laborious. Therefore, workers (especially those handling large flocks) must be strictly supervised as it is alleged that they may neglect hygiene monitoring duties. Apparently, these microbes can then be spread within houses by worker movements, but exposure in birds may also be through air transmission, contaminated water, and pecking of anticipated feed (such as insects) in their environment [25].

It has been acknowledged that host-related factors, such as age, have a large effect on intestinal microbiota [26, 27]. In this study, birds aged 1 month and below were more susceptible to ESBL-/AmpC-EC carriage, and this is because the gut microbiome of these birds is still maturing, making it easy for colonization by various pathogenic bacteria if they exist in the environment. Additionally, due to their upcoming immunity, survival of ingested bad bacteria (such as ESBL/AmpC) through the gastrointestinal tract is also increased if maternal antibodies against them are devoid [28].

Antimicrobial stewardship vices such as misuse of antibiotics during treatment and prophylaxis and for growth promotion are a major cause of the emerging AMR. In our local setting, the bacterial exposure to *β*-lactam antibiotics used for various purposes in poultry farming could spur AMR development. Our research portrayed no use of *β*-lactam derivatives which could be linked to the limited access. Thus, we could not satisfactorily relate the emergence of these strains to usage practices. However, there is evidence of vast use in human health [29] creating likely pathways of development and crossover from people. Although not documented, there are also reports of human drug use in poultry disease management due to (1) lack of access to antibiotics in poor communities and (2) running out of treatment options majorly in nonresponding infections. Information criticizing Ugandan poultry products to be contaminated with *β*-lactam (penicillins, cephalosporins, carbapenems, carbacephems, and monobactams) antibiotic residues could act as a signal for the use of human critical drugs in agriculture. However, these data are lacking which then create an untested hypothesis for our study: could the emergence and spread of ESBL-/AmpC-EC in poultry be chiefly influenced by the pillars of infection prevention and control, particularly animal husbandry practices, biosecurity measures, and vaccinations? All the isolates were lost due to poor biobank management, which hindered our possibility to establish the antibiograms of the ESBL-/AmpC-EC. However, the results still gave a picture on the prevalence and the current forces at work behind the existence of ESBL-/AmpC-EC in local commercial flocks. We did not venture into the possibility of spillover into food chain (through assessing ESBL/AmpC status in poultry products) and apparently healthy humans such as workers in close proximity with the flocks colonized by these zoonotic bacteria. We therefore propose further in-depth studies with a combination of molecular assays (typing ESBL/AmpC genotypes and sequencing) and bioinformatics to explain the ESBL/AmpC public health outcomes, such as transmission dynamics between humans, animals, and the environment.

## 5. Conclusions

This study confirmed that Ugandan poultry can be a potential reservoir of ESBL-/AmpC-EC shed into the environment through fecal matter. The lethal bacteria detected could spread into the food chain through consumption of contaminated meat and eggs, which calls for the institution of stringent food safety systems to minimize transmission to humans. We suggest more risk-based AMR control studies to target intensive broiler farms in different geographical locations. In these flocks, ESBL-/AmpC-EC found is an indicator of potential treatment failures when managing colibacillosis. It is, therefore, important that risky habits such as blind treatments be replaced by embracing accurate diagnostics such as bacterial culture and sensitivity tests so as to avoid AMR emergence, spread, and losses. Sensitization of farmers on proper infection prevention and control measures is also encouraged to reduce likelihood of ESBL-/AmpC-EC introduction and spread in farms.

## Figures and Tables

**Table 1:** Bivariate logistic regression analysis: factors related to ESBL-/AmpC-EC carriage among commercial chickens.

Variable	*N*	ESBL/AmpC status	Bivariate analysis	
		Yes (*n*_1_, %)	*p* value	COR; 95% CI
*Flock size*				
<700^*∗*^	140	34 (24.3)	0.010	2.32; 1.23–4.39
700–1,200^*∗*^	60	15 (25.0)	0.026	2.41; 1.11–5.23
1,201–1,701	60	4 (6.7)	0.254	0.52; 0.17–1.61
>1,701	140	17 (12.1)	a	1.00; ref

*Age*				
<1 month^*∗*^	44	11 (25.0)	0.007	6.33; 1.65–24.35
1–4 months^*∗*^	196	43 (21.9)	0.007	5.34; 1.59–17.89
5–8 months	100	13 (13.0)	0.115	2,84; 0.77–10.41
>8 months	60	3 (5.0)	a	1.00; ref

*Type of commercial chicken*				
Broilers^*∗*^	240	64 (26.7)	<0.001	9.82; 3.85–25.07
Dual purpose	20	1 (5.0)	0.754	1.42; 0.16–12.83
Layers	140	5 (3.6)	a	1.00; ref

*Chicken housing system*				
Battery cage	20	0 (0.0)	0.998	a
Deep litter	380	70 (18.4)	a	1.00, ref

*Subcounty*				
Kakiri	100	12 (12.0)	a	1.00; ref
Kasangati Town Council	100	18 (18.0)	0.238	1.61; 0.73–3.55
Kyengera Town Council	100	14 (14.0)	0.674	1.19; 0.52–2.73
Mende	40	9 (22.5)	0.121	2.13; 0.82–5.54
Wakiso	20	1 (5.0)	0.374	0.39; 0.05–3.15
Wakiso Town Council^*∗*^	40	16 (40.0)	<0.001	4.89; 2.04–11.72

COR: crude odds ratio; CI: confidence interval; ref: reference group; a: no statistic computed. ^*∗*^Significant factor by bivariate analysis.

## Data Availability

The datasets used to support the findings of this study are available from the corresponding author upon request.

## References

[1] Dierikx C. M., van der Goot J. A., Smith H. E., Kant A., Mevius D. J. (2013). Presence of ESBL/AmpC-producing Escherichia coli in the broiler production pyramid: a descriptive study. *PLoS One*.

[2] Gazal L. E. d. S., Medeiros L. P., Dibo M. (2021). Detection of ESBL/AmpC-producing and fosfomycin-resistant Escherichia coli from different sources in poultry production in Southern Brazil. *Frontiers in Microbiology*.

[3] Ndoboli D., Nganga F., Lukuyu B. (2021). The misuse of antiretrovirals to boost pig and poultry productivity in Uganda and potential implications for public health. *International Journal of One Health*.

[4] Brolund A. (2014). Overview of ESBL-producing enterobacteriaceae from a nordic perspective. *Infection Ecology & Epidemiology*.

[5] Aliyu A. B., Saleha A. A., Jalila A., Zunita Z. (2016). Risk factors and spatial distribution of extended spectrum *β*-lactamase-producing-Escherichia coli at retail poultry meat markets in Malaysia: a cross-sectional study. *BMC Public Health*.

[6] Daehre K., Projahn M., Friese A., Semmler T., Guenther S., Roesler U. H. (2018). ESBL-producing *Klebsiella pneumoniae* in the broiler production chain and the first description of ST3128. *Frontiers in Microbiology*.

[7] da Silva M. V. (2013). *Poultry and Poultry Products—Risks for Human Health*.

[8] Kakooza S., Muwonge A., Nabatta E. (2021). A retrospective analysis of antimicrobial resistance in pathogenic Escherichia coli and Salmonella spp. isolates from poultry in Uganda. *International Journal of Veterinary Science and Medicine*.

[9] Lammie S. L., Hughes J. M. (2016). Antimicrobial resistance, food safety, and one health: the need for convergence. *Annual Review of Food Science and Technology*.

[10] Huijbers P. M. C., de Kraker M., Graat E. A. M. (2013). Prevalence of extended-spectrum *β*-lactamase-producing Enterobacteriaceae in humans living in municipalities with high and low broiler density. *Clinical Microbiology and Infections*.

[11] Brower C. H., Mandal S., Hayer S. (2017). The prevalence of extended-spectrum beta-lactamase-producing multidrug-resistant Escherichia coli in poultry chickens and variation according to farming practices in Punjab, India. *Environmental Health Perspectives*.

[12] Hosuru Subramanya S., Bairy I., Nayak N. (2020). Detection and characterization of ESBL-producing enterobacteriaceae from the gut of healthy chickens, gallus gallus domesticus in rural Nepal: dominance of CTX-M-15-non-ST131 Escherichia coli clones. *PLoS One*.

[13] Aworh M. K., Kwaga J., Okolocha E. (2020). Extended-spectrum *ß*-lactamase-producing Escherichia coli among humans, chickens and poultry environments in Abuja, Nigeria. *One Heal Outlook*.

[14] Chishimba K., Hang’ombe B. M., Muzandu K. (2016). Detection of extended-spectrum beta-lactamase-producing Escherichia coli in market-ready chickens in Zambia. *International Journal of Microbiology*.

[15] Odoch T., Wasteson Y., L’Abée-Lund T. (2017). Prevalence, antimicrobial susceptibility and risk factors associated with non-typhoidal salmonella on Ugandan layer hen farms. *BMC Veterinary Research*.

[16] Wang X., Cheng Z. (2020). Cross-sectional studies: strengths, weaknesses, and recommendations. *Chest*.

[17] FAO Livestock production systems spotlight: Uganda chicken meat and beef.

[18] Jacob M. E., Keelara S., Aidara-Kane A., Matheu Alvarez J. R., Fedorka-Cray P. J. (2020). Optimizing a screening protocol for potential extended-spectrum *β*-lactamase Escherichia coli on MacConkey agar for use in a global surveillance program. *Journal of Clinical Microbiology*.

[19] Olsen R. H., Bisgaard M., Löhren U., Robineau B., Christensen H. (2014). Extended-spectrum *β*-lactamase-producing Escherichia coli isolated from poultry: a review of current problems, illustrated with some laboratory findings. *Avian Pathology*.

[20] Nilsson O., Börjesson S., Landén A., Greko C., Bengtsson B. (2020). Decreased detection of ESBL-or pAmpC-producing Escherichia coli in broiler breeders imported into Sweden. *Acta Veterinaria Scandinavica*.

[21] Nakayama T., Ueda S., Huong B. T. M. (2015). Wide dissemination of extended-spectrum *β*-lactamase-producing Escherichia coli in community residents in the Indochinese peninsula. *Infection and Drug Resistance*.

[22] Sarba E. J., Kelbesa K. A., Bayu M. D., Gebremedhin E. Z., Borena B. M., Teshale A. (2019). Identification and antimicrobial susceptibility profile of Escherichia coli isolated from backyard chicken in and around ambo, Central Ethiopia. *BMC Veterinary Research*.

[23] Nguyen V. T., Jamrozy D., Matamoros S. (2019). Limited contribution of non-intensive chicken farming to ESBL-producing Escherichia coli colonization in humans in Vietnam: an epidemiological and genomic analysis. *Journal of Antimicrobial Chemotherapy*.

[24] Rolain J.-M. (2013). Food and human gut as reservoirs of transferable antibiotic resistance encoding genes. *Frontiers in Microbiology*.

[25] Hedman H. D., Vasco K. A., Zhang L. (2020). A review of antimicrobial resistance in poultry farming within low-resource settings. *Animals*.

[26] Kers J. G., Velkers F. C., Fischer E. A. J., Hermes G. D. A., Stegeman J. A., Smidt H. (2018). Host and environmental factors affecting the intestinal microbiota in chickens. *Frontiers in Microbiology*.

[27] Yegani M., Korver D. R. (2008). Factors affecting intestinal health in poultry. *Poultry Science*.

[28] Cosby D. E., Cox N. A., Harrison M. A., Wilson J. L., Buhr R. J., Fedorka-Cray P. J. (2015). Salmonella and antimicrobial resistance in broilers: a review. *Journal of Applied Poultry Research*.

[29] National Drug Authority (2020). *National Drug Register of Uganda Human Medicines*.

